# The National Pension Fund

**Published:** 1887-07-09

**Authors:** 


### THE NATIONAL PENSION FUND.

It will save a good deal of trouble if nurses and other hospital officials who propose joining the National Pension Fund will send their names and other particulars to *the Secretary of The Hospitals Association*, Norfolk House, Norfolk Street, W.C., and not to the Editor of The Hospital. Names continue to arrive in considerable numbers, but if those interested desire the Fund to be started before the end of the Jubilee year they must influence and interest all their friends immediately.

---

Nurses and other hospital officials are beginning to understand clearly that in sending their names they are not committing themselves in any way. They are merely expressing their willingness to join a Pension Fund if its details be such as their judgment can approve. It would be well if the Fund could be definitely started before the expiration of the Jubilee year. This can only be done by those who propose to join it. If any are enthusiastically in favour of it, they might circulate among their nurse friends half a dozen weekly copies of The Hospital, calling special attention to the Fund. It is for nurses and other officials that the pensions are designed, and they should rouse themselves and one another to earnest effort in order to bring the matter speedily into working order.

---

From a Trained Nurse of the London Association of Nurses, 62, New Bond Street, W.

Hoping that you will permit monthly nurses to join your Pension Fund, if they can show that they are fully qualified, I venture to send my own and my sister's names; we belong to the London Association of Nurses. I also send you the name of another sister, who is at the Evelina Hospital. As you invite suggestions, I think monthly nurses and private nurses should pay at least double the entrance fee or subscription that those engaged in hospital work pay. Wishing the movement all success.

---

The Lady Superintendent of a Provincial Institution informs us that she will gladly place her name on the Pension List. She has four nurses and four probationers, but only one nurse receives £25 per annum. The other three nurses have gone on as nurses from probationers, and none of them have been over two years, consequently they do not yet receive full pay, and cannot afford £5 a year. She then proceeds:—"Will you answer me a few questions? Can one pay less than £5 per annum? How many years must one pay before one can claim a pension? Because nurses of twenty-five or twenty would not, I am sure, care to pay in £5 per annum if they could not receive a pension before they are fifty years of age. Supposing one breaks down before fifty, can one claim? If one dies, can a relation claim any of the money that has been paid in? Nurses will often put away a little in a savings-bank or sick-club, because then they can, in the one case, draw if ill, in the other, if they require it."

---

The following is a list of the sixty-nine Institutions from which names have been received by Mr. Howard J. Collins, in support of the formation of a "National Pension Fund for Hospital Officials and Trained Nurses." The figures in parentheses denote the number of names received to 30th June, 1887.

Guy's Hospital (1).
St. Mary's Hospital (1).
Miller Hospital, Greenwich (5).
Cancer Hospital, Brompton (1).
Hospital for Consumption, Brompton (1).
Western Hospital, Fulham (Met. Asylums Board) (3).
Foundling Hospital (1).
North Eastern Hospital for Children (1).
R. N. H., Haslar, Gosport (1).
Dewsbury General Infirmary (4).
Isle of Man Hospital (1).
Lowestoft Hospital (2).
Yeovil Hospital (1).
R. S. Hospital, Ottenshaw, Chertsey (1).
Royal Alexandra Hospital for Children, Brighton (1).
Essex and Colchester Hospital (1).
Lancaster Infirmary (6).
Torbay Hospital (9).
Monsall Hospital, Manchester (2).
Grimsby Hospital (1).
Swansea Hospital (1).
Hangleton Hospital, Portslade, near Brighton (1).
Macclesfield Infirmary (1).
Wolverhampton and Staffordshire General Hospital (2).
Windsor Royal Infirmary (3).
Oldham Infirmary (1).
West Bromwich District Hospital (1).
Royal Infirmary, Glasgow (1).
Royal Berks Hospital (1).
Beckett Hospital, Barnsley (1).
Victoria Hospital for Barnsley and District (9).
St. Marylebone Infirmary (1).
Hampstead Workhouse Infirmary (19).
Manchester Workhouse Infirmary (1).
City Poor House Hospital, Aberdeen (1).
Earlswood Asylum (1).
Llandudno Cottage Hospital (1).
Totnes Cottage Hospital (2).
Eastry Cottage Hospital (1).
Erith Cottage Hospital (1).
Enfield Cottage Hospital (2).
Bridport Cottage Hospital (1).
Moreton-in-Marsh Cottage Hospital (2).
Burton-on-Trent Cottage Hospital (2).
Blackheath and Charlton Cottage Hospital (1).
Watford District Cottage Hospital (1).
Boston Cottage Hospital (3).
Beechwood Convalescent Home (1).
Llandrindod Wells Convalescent Home (2).
Workhouse Infirmary Nursing Association (1).
Hampshire Nurses' Institution (16).
Belgravia Institution for Trained Nurses (1).
The Nursing Institution, Henrietta Street, Covent Garden (69)
Leeds Nurses' Home (9).
Private Nursing Establishment, Glasgow (1).
The Nursing Home, London Hospital, E. (1).
Highbury Association of Trained Nurses (9).
Cambridge Nurses' Home (1).
Birkenhead Nurses' Institution (1).
Aberfeldy Nurses' Home (1).
Eastbourne Nurses' Institution (1).
Cobham Nursing Home (1)
Southport Nurses' Home (1).
London Association of Nurses, New Bond Street (5).
County of Cornwall Trained Nurses' Home, Truro (1).
Norfolk and Norwich Staff of Hospital Trained Nurses (26).
Hampstead Home Hospital and Nursing Institute (14).
London Orphan Asylum Infirmary (2).
Mission House, Commercial Road, E. (1).

Thirty names have also been received from Private Nurses and others who do not mention the name of any Institution with which they may be connected.

Inquiries have been made on behalf of the following Institutions, but no names in support of the scheme have yet been received.

The Nightingale Fund.
West London Hospital.
Evelina Hospital for Sick Children.
Sheffield General Infirmary.
East Park Home Infirmary for Children, Glasgow.
Establishment for Invalid Gentlewomen.
Leicester Nurses' Home.
Salisbury Nurses' Home.
Wolverhampton Nursing Institution.
Wigmore Street Institution for Trained Nurses.

